# Intracranial Tuberculoma in a Pregnant Lady: A Hitherto Unknown Case and a Successful Outcome

**DOI:** 10.7759/cureus.31772

**Published:** 2022-11-22

**Authors:** Roopeessh Vempati, Periyaiyadever Samuganathan, Prithvi Raghavan, Shreyaa Rajpal, Chinmay Guralwar, Bhavani Padamati, Tamara Tango, Sweta Sahu, Thrilok Chander Bingi

**Affiliations:** 1 Internal Medicine, Gandhi Medical College and Hospital, Hyderabad, IND; 2 General Practice, Impian Care Clinic, Kuala Lumpur, MYS; 3 Internal Medicine, Osmania Medical College, Hyderabad, IND; 4 Community Medicine, Government Medical College and Hospital, Nagpur, IND; 5 Internal Medicine, Atlantic University School of Medicine, Rodney Bay, LCA; 6 Neurology, Faculty of Medicine Universitas Indonesia, Jakarta, IDN; 7 Surgery, Jagadguru Jayadeva Murugarajendra (JJM) Medical College, Davanagere, IND; 8 General Medicine, Gandhi Hospital, Hyderabad, IND

**Keywords:** tuberculosis, central nervous system tuberculosis, cns-tb, intracranial tuberculoma, neuroimaging, tubal infertility, tuberculoma, pregnant, seizures

## Abstract

Central nervous system tuberculosis (CNS-TB) is rarely suspected in pregnancy because its clinical presentation may masquerade other common conditions in pregnancy, such as eclampsia. In high tuberculosis endemic areas, CNS-TB should be suspected with a high degree of suspicion among unimmunized and immunocompromised individuals. We hereby report a case of a 32-year-old pregnant woman conceived by in vitro fertilization due to tubal blockage causing infertility, probably due to chronic infection, who presented with a history of multiple seizure episodes without a history of similar complaints outside this pregnancy. Obstetric examination revealed a gravid uterus larger than the corresponding gestational age, and an antenatal scan confirmed dichorionic diamniotic twins with the first twin in the breech and the second twin in the cephalic presentation. Magnetic resonance imaging of the brain revealed multiple nodular lesions of varying sizes that were isointense on T1-weighted imaging and hypointense on T2-weighted imaging in multiple regions of the brain, which suggest tuberculomas. A preterm cesarean section was performed at 31 weeks gestational age due to preterm rupture of membranes. We report this case to enlighten the physicians in diagnosing seizures causing intracranial tuberculoma in pregnant women and utilizing the role of imaging in diagnosis.

## Introduction

Extrapulmonary tuberculosis (EPTB) is a clinical manifestation of tuberculosis (TB) in organs other than the lungs. EPTB accounts for 20-30% of all cases of TB, which affects mainly children and immunocompromised patients [1]. One of the target organs of *Mycobacterium tuberculosis* is the central nervous system, which causes a disease known as central nervous system tuberculosis (CNS-TB). CNS-TB constitutes about 2-5% of all TB cases [2]. CNS-TB consists of tuberculous meningitis (TM), tuberculomas, and tuberculous spinal arachnoiditis. CNS-TB may occur among pregnant women with similar pathogenesis as those in non-pregnant women. The establishment of a CNS-TB diagnosis is challenging among pregnant women [3]. TB in pregnancy might present insidiously with malaise and fatigue, which can be taken for granted as normal pregnancy symptoms [4]. The symptoms of CNS-TB may mimic other conditions related to pregnancy, such as pre-eclampsia, seizure disorder, hyperemesis, and brain tumors [3]. Previously, it has been reported regarding the atypical presentations of TM in pregnancy [5]. Therefore, the clinical presentation of CNS-TB in pregnancy needs to be explored. We present a case of intracranial tuberculoma in a successful twin pregnancy.

## Case presentation

A 32-year-old pregnant woman, G1P0A0, with 27 weeks of gestational age, presented with recurrent generalized tonic-clonic seizures since 21 weeks of gestational age. She was conceived by in vitro fertilization (IVF) due to tubal blockage causing infertility. On eliciting the history, this patient had similar episodes in the 21st and 23rd weeks of gestation and no similar complaints outside this pregnancy. There was no history of a head injury, fever, neck stiffness, or vomiting. The history of gestational hypertension and hypertension outside this pregnancy was denied. This patient also did not experience shortness of breath, cough, cold, chest pain, hemoptysis, loss of weight, or loss of appetite in the past. The patient was never diagnosed with epilepsy or pulmonary TB. However, this patient was known to have had close contact with a pulmonary TB patient. She did not have a prior medical check-up after exposure to a TB patient.

The patient underwent cervical cerclage at 12 weeks gestational age for cervical incompetence. The family history of epilepsy or seizures was unknown. The patient was not immunized with the bacillus Calmette-Guérin (BCG) vaccination. Regarding the eating habit of the patient, she did not consume undercooked meat. The patient was E4V2M6 and had somnolence. The vital signs of the patient on admission were a blood pressure of 110/80 mmHg, 80 beats per minute for the heart, 18 cycles per minute for the respiratory rate, a saturation of 98% for oxygen, and she was afebrile. The obstetric examination revealed a gravid uterus whose size corresponds to greater than gestational age (30 weeks), with two fetal heads palpable, one in the breech position and the other in the cephalic presentation. A vaginal examination revealed a cervical stitch in situ. The neurological and ophthalmological examinations were within normal limits. The results of the laboratory investigation and cerebrospinal fluid analysis are shown in Tables 1, 2.

**Table 1:** Laboratory investigation results AST: aspartate transaminase; ALT: alanine transaminase; ALP: alkaline phosphatase; HIV: human immunodeficiency virus; HBsAg: hepatitis B surface antigen; HCV: hepatitis C virus; AFB: acid-fast bacilli; Ig: immunoglobulin.

Laboratory investigation	Results	Normal references
Complete blood count		
Hemoglobin (g/dL)	11	10-14
Red blood cell count (mill/mm^3^)	4	3.5-4.5
White blood cell count (per mm^3^)	14,000	9,000-16,000
Platelet count (lakh per mm^3^)	1.84	1.5-4.3
Red cell distribution width (%)	20	11-5
Mean corpuscular volume (fL)	90	81-96
Neutrophils (%)	75	40-70
Lymphocytes (%)	15	20-45
Monocytes (%)	8.7	2-8
Eosinophils (%)	1.2	1-6
Basophils (%)	0.3	0-1
Liver function test		
Total bilirubin (mg/dL)	0.29	0.3-1.2
Direct bilirubin (mg/dL)	0.21	<0.2
AST (U/L)	19	<35
ALT (U/L)	7	<35
ALP (U/L)	173	38-126
Albumin (g/dL)	2.11	3.5-5.2
Renal function test		
Blood urea (mg/dL)	14	17-43
Creatinine (mg/dL)	0.57	0.55-1.2
Uric acid (mg/dL)	2.3	2.6-6
Erythrocyte sedimentation rate	140 mm in the first hour and 180 mm in the second hour	<30 mm in the first hour and <60 mm in the second hour
C-reactive protein (μg/mL)	21	0.4-20.3
Blood grouping and typing	A negative	
HIV	Negative	
HBsAg	Non-reactive	
HCV	Non-reactive	
Mantoux test	Negative	
Sputum for AFB (Ziehl-Neelsen staining)	Acid-fast bacilli not detected	
Sputum for culture	Acid-fast bacilli not grown	
IgM and IgG toxoplasma antibody	Negative	
Stool microscopy	Negative for parasites and oocytes	
Direct and indirect Coombs tests	Negative	

**Table 2:** Cerebrospinal fluid analysis CSF: cerebrospinal fluid; ADA: adenosine deaminase; CBNAAT: cartridge-based nucleic acid amplification test.

Laboratory investigation	Results	Normal references
Cerebrospinal fluid protein (mg/dL)	21	15-45
Cerebrospinal fluid glucose (mg/dL)	31	50-80
CSF ADA (U/L)	6	<10
CSF CBNAAT	Negative	

**Figure 1:**
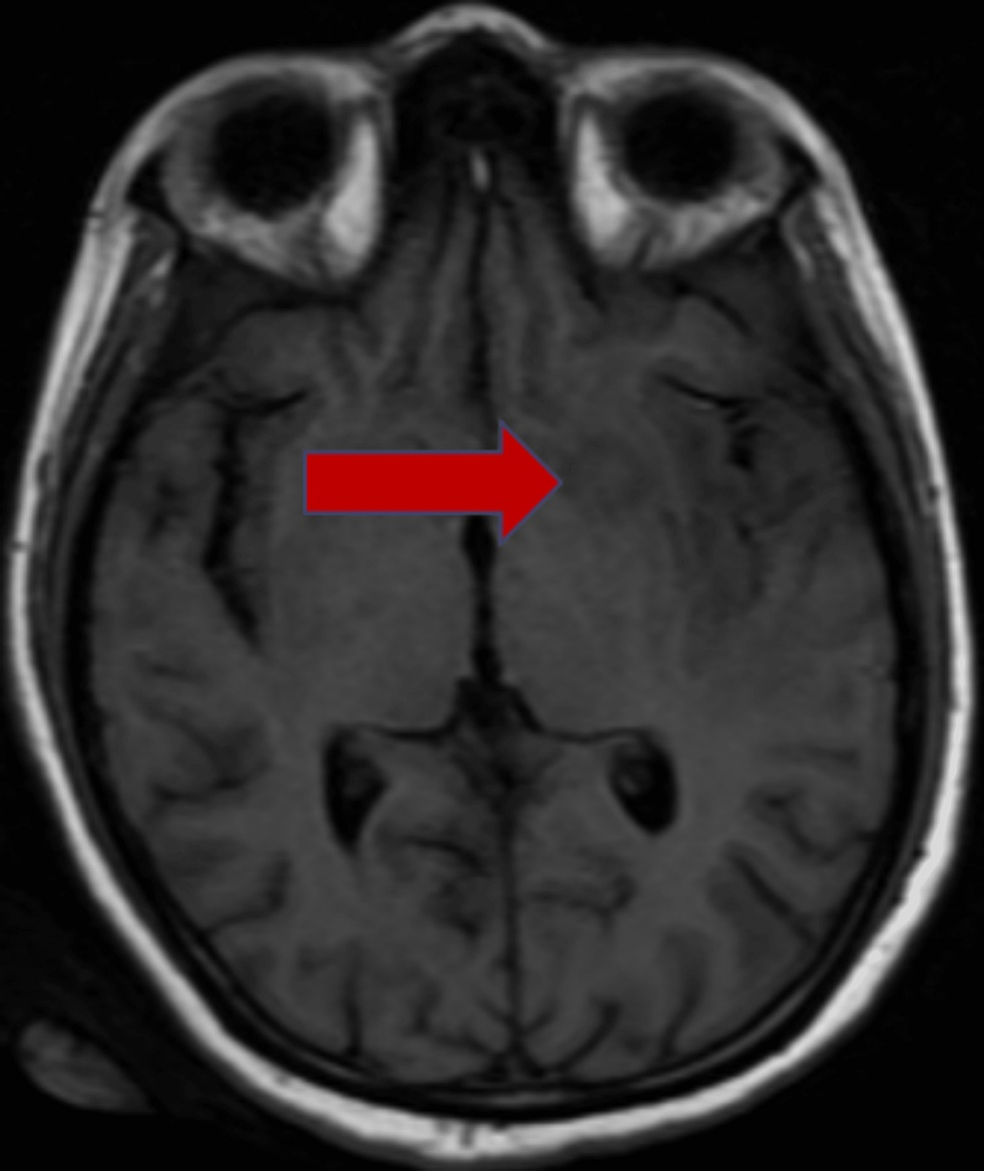
T1-weighted MRI of the brain in axial projection The red arrow marks an isointense ring lesion in the left capsuloganglionic region.

**Figure 2:**
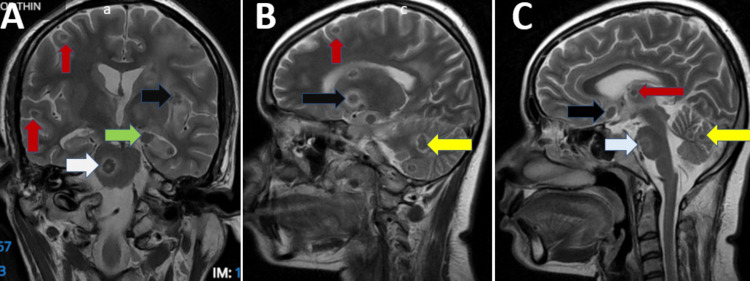
T2-weighted MRI of the brain showing coronal (A) and sagittal (B, C) projection Hypointense ring-enhancing lesions marked with arrows in (A) gray-white matter junction (red), right pons (white), conglomerate lesions near the left insular cortex (black), and left hippocampus (green); (B) cerebellum (yellow), gray-white matter junction (red), and basal ganglia (black); and (C) thalamus (red), medial temporal lobe near the optic chiasm (black), pons (white), and cerebellum (yellow).

**Figure 3:**
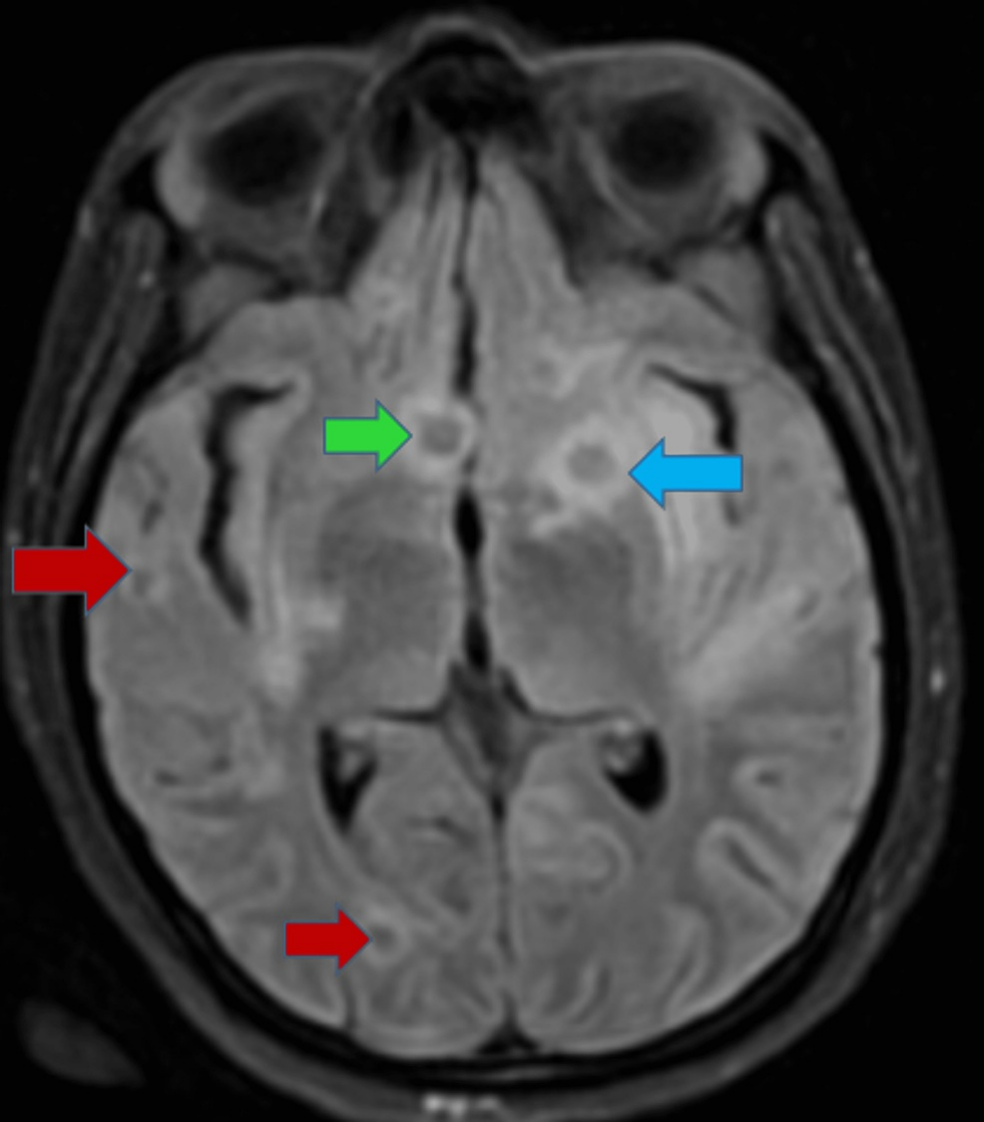
FLAIR MRI of the brain in axial projection Hypointense ring-enhancing lesions are represented by arrows at the gray-white matter junction (red), left capsuloganglionic region (blue), and medial temporal lobe (green). FLAIR: fluid-attenuated inversion recovery.

**Figure 4:**
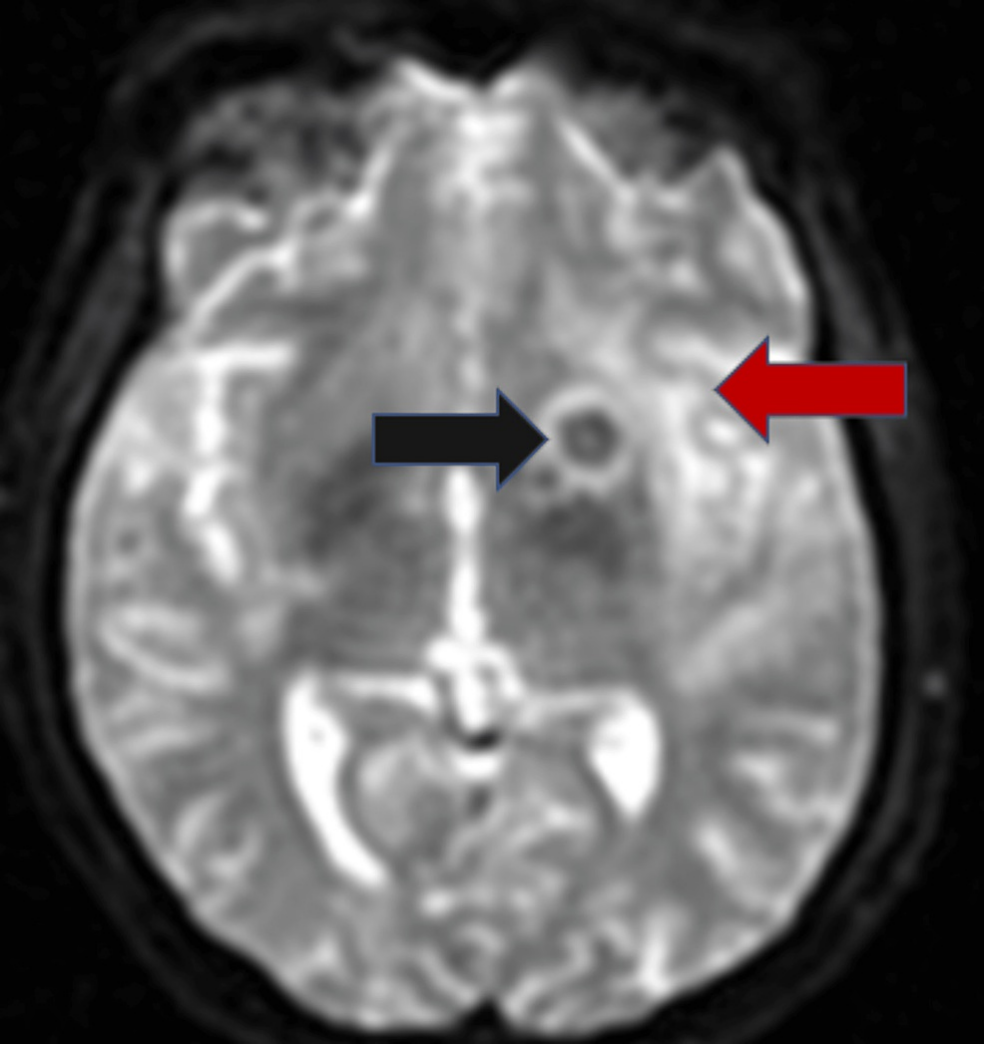
Diffusion-weighted imaging of T2-weighted MRI of the brain in axial projection The black arrow represents tuberculoma showing no foci of restriction on diffusion-weighted imaging. The red arrow represents a hyperintense area in the left perisylvian cortex showing foci of diffusion restriction, likely post-ictal edema.

**Figure 5:**
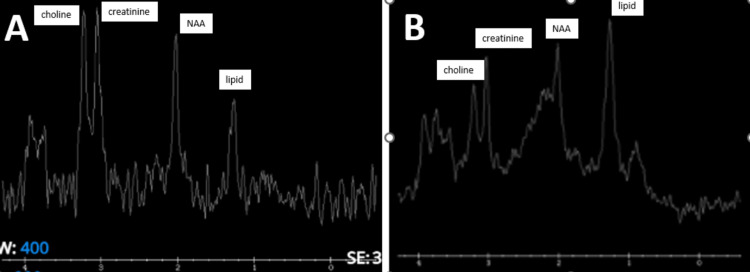
Magnetic resonance spectroscopy of ring lesions (A) Increased choline creatinine ratio, decreased NAA, and increase in lipid. (B) Decreased NAA and lipid peak. NAA: N-acetyl aspartate.

An antenatal scan was performed at admission and revealed an intrauterine twin pregnancy and dichorionic diamniotic, with the fetus in breech presentation and cephalic presentation for the first and second fetuses, respectively. The MRI scan revealed multiple nodular lesions of varying sizes that were isointense on T1-weighted imaging (Figure 1) and hypointense on T2-weighted imaging (Figure 2) and fluid-attenuated inversion recovery (FLAIR; Figure 3), with minimal perilesional edema in bilateral cerebral hemispheres predominantly in the gray-white matter junction, bilateral capsuloganglionic region, right pons, bilateral cerebellar hemispheres, vermis, and left thalamus, as well as the hippocampus. On diffusion-weighted imaging (DWI), it showed tuberculoma without foci of restriction (Figure 4). Magnetic resonance spectroscopy (MRS) of the patient showed an increased choline-creatinine ratio, decreased N-acetyl aspartate (NAA), and a lipid peak (Figure 5). The fetal Doppler showed a normal study and no uteroplacental or fetoplacental insufficiency. Abdominal ultrasonography (USG) and pelvic USG were normal. The two-dimensional echocardiography was normal.

Management

The patient was admitted to the obstetric intensive care unit. She was initially treated with 2 mg intravenous midazolam as needed and 1,000 mg intravenous levetiracetam BID (bis in die - twice a day) for subsiding the seizure episodes and on 500 mg intravenous levetiracetam BID for maintenance. This patient was also given 6 mg intravenous dexamethasone BID and 1,000 mg intravenous ceftriaxone BID.

The diagnosis of gestational hypertension was established due to hypertensive episodes, indicated by blood pressure >/= 140/90 on multiple occasions in blood pressure monitoring. The patient was administered 20 mg intravenous labetalol as needed, and the patient was put on maintenance using 100 mg labetalol per oral BID. The patient was started on antituberculous therapy with a fixed drug regimen administered orally three times a day consisting of 75 mg isoniazid, 150 mg rifampicin, 400 mg pyrazinamide, 275 mg ethambutol, and 40 mg pyridoxine per oral OD (once a day) after MRI findings suggested intracranial tuberculoma.

While in the intensive care unit, she developed multiple episodes of breakthrough seizures with clonic movements of the right upper and lower limbs, associated with head movements, and preserved awareness. The diagnosis of focal status epilepticus was established. The patient was shifted to acute medical care, where she was managed with 2 mg intravenous midazolam as needed, 500 mg intravenous sodium valproate BID, and an escalated dose of levetiracetam (750 mg intravenous levetiracetam BID), even after which she had breakthrough seizures with right upper and lower limb clonic movements. A dose of 4 mg intravenous lorazepam as needed was added, and the intravenous levetiracetam dose was escalated to 1,000 mg BID, after which seizures subsided. Then 4 mg of intravenous lorazepam was de-escalated to 200 mg of lacosamide per oral dose and 5 mg of clobazam per oral HS (hora somni - take at bedtime) dose. A 12.5 mg dose of metoprolol per oral OD was added for gestational hypertension, along with 100 mg of labetalol per oral BID. Eventually, the patient was de-escalated to 500 mg levetiracetam per oral BID, 500 mg sodium valproate per oral BID, along with 50 mg lacosamide per oral BID, and 5 mg clobazam per oral HS, as seizure episodes had subsided. A 6 mg intravenous dexamethasone BID was given for a month and then tapered gradually until being stopped.

The patient was planned for a preterm cesarean section at 31 weeks of gestational age because of a preterm rupture of membranes. She delivered an alive female weighing 1,400 grams with no gross congenital anomaly and an alive male weighing 1,500 grams with no gross congenital anomaly. Later, the cervical stitch was removed. Both babies were found to be Rh-positive. Then the mother was given anti-D immunoglobulin on postoperative day one. Babies were admitted to the neonatal intensive care unit for preterm care.

In the postpartum period, this patient developed involuntary movements of the right upper limb, which were presumed to be choreoathetotic type, and 0.5 mg of clonazepam per oral BID was prescribed while clobazam was discontinued. As the movements did not subside, tetrabenazine per oral was advised. However, this patient denied tetrabenazine. Then the patient was discharged with antituberculous therapy, pyridoxine tablets, levetiracetam tablets, sodium valproate tablets, lacosamide tablets, labetalol tablets, and metoprolol tablets. The patient was given awareness regarding the excretion of drugs in breast milk and was advised to not stop breastfeeding while being compliant with medication due to the usefulness of breast milk to the infants and also the importance of medication for the mother.

## Discussion

TB is the most common cause of mortality in women of reproductive age when compared to maternal mortality due to all other causes combined. The prevalence of TB in pregnant women is considered to be the same as that in the general population since the exact number of pregnant women infected with TB is unknown. TB in pregnancy can cause significant maternal and perinatal morbidity, with 2.6 million cases out of 11 million worldwide prevalent in India. Malnutrition, immunosuppressed status, and other comorbidities affect the disease course, which leads to a flare [6]. Besides that, suppression of Th1 in pregnancy leads to increased infection susceptibility and TB reactivation [7].

Pelvic TB can cause salpingitis, endometritis, cervicitis, and irregular vaginal bleeding in non-pregnant women [7]. Compared to CNS-TB, pelvic TB is more common in India, leading to infertility in one-third of cases [6]. Our patient had a history of infertility due to a tubal block, for which she underwent IVF. We suspect infertility in this patient was due to pelvic TB.

Diagnosing TB in pregnancy is challenging. Not only because clinical features are attributed to normal pregnancy symptoms, but also because the weight gain associated with pregnancy masks the weight loss due to TB [6]. Although the patient did not experience weight loss, she experienced malaise and lassitude. TB in pregnancy can lead to spontaneous miscarriage, low baby birth weight, preterm labor, increased neonatal mortality risk, and congenital TB. Rarely, TB can cause high perinatal mortality and affect 66% of pregnancies in TB-infected pregnant women [6,7].

The diagnosis of cerebral tuberculoma in this pregnant patient was supported by the fact that the patient was in close contact with a pulmonary TB patient. In addition, the laboratory finding that supported the diagnosis of CNS-TB in this patient was the low cerebrospinal fluid glucose level. However, we did not observe other typical laboratory findings in this patient, such as an elevated cerebrospinal fluid protein level, the presence of CSF adenosine deaminase (ADA), or the finding of acid-fast bacilli (AFB) on sputum for AFB (Ziehl-Neelsen staining) [8], which is the unusual feature of our case.

Computed tomography (CT) has been used to identify tuberculoma owing to its wide availability. Compared to a CT scan, an MRI is preferable due to its higher resolution and better visualization of the posterior fossa. MRI techniques, such as DWI, increase accuracy in identifying tuberculoma [9]. According to the literature, tuberculoma is ring enhancing with gadolinium contrast MRI, which needs to be differentiated from other ring-enhancing lesions. The differential diagnosis for ring-enhancing lesions on MRI includes cerebral abscess, cerebral metastasis, cerebral toxoplasmosis, and primary CNS lymphoma. Lesions of the cerebral abscess may be single or multiple, occurring at gray-white matter junctions along the middle cerebral artery [10]. Cerebral metastasis can be homogeneously enhanced, occurring at gray-white matter junctions and watershed zones [10]. Cerebral toxoplasmosis can be characterized by single or multiple lesions that occur at the basal ganglia and corticomedullary junction [10]. Cerebral toxoplasmosis was excluded as the patient did not consume undercooked meat and the laboratory finding showed negative IgM and IgG toxoplasma antibodies. Primary CNS lymphoma ring enhancement is uncommon and may occur in the periventricular white matter or corpus callosum [10]. Though MRI with contrast was not done in view of pregnancy, our diagnosis is supported by history and clinical correlation. Another possible differential diagnosis is neurocysticercosis. Neurocysticercosis may have seizures as its clinical presentation. Neurocysticercosis may occur in people who do not even eat pork and have no contact with pigs. Although humans play a role as the final host, humans may sometimes become the intermediate host by ingesting ova shed in the feces of a human carrier. In addition, cysticercosis may develop in tapeworm carriers through autoinfection. Neurological sequelae followed by subsequent larvae may settle in the brain, and more rarely, the spinal cord [11].

MRS is useful to diagnose cerebral tuberculoma. Studies have reported that MRS showed a specific lipid peak in cases of tuberculoma. Lipid peaks are observed at 0.9, 1.3, 2.0, and 2.8 ppm. Lipid resonances at 0.9 and 1.3 ppm are assigned to methylene groups and terminal methyl groups of fatty acids found within the center of tuberculoma. Tuberculomas have increased choline-creatinine ratio, decreased NAA, and lipid peak; this is in line with findings in our case. The TB evidence may be supported by other imaging findings on other parts of the body, such as the lungs and lymph nodes. Signs and symptoms, such as fever and a raised erythrocyte sedimentation rate, may also be present to suggest an inflammatory lesion, although they are not specific or confirmatory. Therefore, MRS would increase the possibility of a correct diagnosis as it can provide additional biochemical information and diffusion imaging that is based on the restriction of water molecules by the lesion [12].

Regarding the treatment, the first-line management for intracranial tuberculoma comprises antituberculous therapy and corticosteroids. Corticosteroids are indicated in cases where there is a paradoxical increase in size while the patients are on antituberculous therapy, and they experience symptoms due to perilesional edema. Conventionally, antituberculous therapy is recommended until perilesional enhancement is resolved. However, most clinicians do not recommend it beyond nine to 12 months [8]. Unfortunately, we did not follow up with the patient until the patient finished antitubercular therapy regimens, which contributed to the limitations of our study. Also, resection of lesions through surgery is thought of in cases like refractory seizures and significant mass effects with or without hydrocephalus [8].

## Conclusions

Identifying the underlying cause of seizures in our patient was crucial, as it helped the physicians initiate definitive management. In our case, even though typical CNS-TB findings are absent, a history of close contact with a pulmonary TB patient, tubal blockage causing infertility in a high-TB endemic area, and a tuberculoma finding on MRS have aided us in clinching the diagnosis. A holistic approach led to successful twin pregnancy outcomes and also saved the life of the mother. Our patient was advised to be strictly compliant with her regimen and discharged to follow up after six weeks. At follow-up, she was found to be seizure free and was encouraged to continue the treatment.
